# West Texas Medical Association

**Published:** 1901-12

**Authors:** W. B. Russ

**Affiliations:** Secretary


### West Texas Medical Association.

The West Texas Medical Association held its twenty-fifth annual meeting in San Antonio on Thursday, October 31, 1901.

The morning session was called to order at 10 o'clock by President Alonzo Garwood, of New Braunfels.

Roll called by Secretary Dinwiddie, which showed the largest attendance in the history of the association.

The members were much gratified to have as visitors former first vice-president of the American Medical Association, Dr. Willis P. King, Dr. Hambleton, of the United States Marine Hospital Service, and the able secretary of the State Board of Medical Examiners and editor of the *Texas Medical News,* Dr. M. M. Smith.

Dr. G. L. Moody read a paper on "Locomotor Ataxia," and presented a case for study, which exhibited, in addition to the classical symptoms of locomotor ataxia, evidence of profound mental disturbance.

Dr. M. L. Graves said: "The complication of locomotor ataxia with a mental disease is to me an extremely interesting subject. We find that Dr. Moody's patient was a sufferer for some months with the lancinating pains of locomotor ataxia before the development of his mental disorder. It is interesting to note that the patient's intellect responds to the suggestion that his hallucinations are imaginary; still, he finds himself compelled to believe in them."

PAPER: "Paresis," by Dr. M. L. Graves, superintendent Southwestern Insane Asylum.

Dr. Kingsley said: "I believe neglected syphilis is responsible for the development of most cases of paresis. Treatment for syphilis, therefore, I think should never be permanently discontinued."

Dr. Hicks: "Some years ago I had a syphilitic patient who exhibited all of the symptoms of advanced paresis. Vigorous antisyphilitic treatment was followed by a rapid disappearance of all of the symptoms, and now for five years the man has been apparently well and at work."

Dr. Lankford: "Paresis occurs chiefly among brain workers, hence in the higher walks of life. I do not believe syphilis to be responsible for the majority of cases. Illiterate individuals seem to be peculiarly immune. In Mexico, for example, where an astonishly large portion of the population are syphilitic, and where brain exhaustion from mental strain is rare, paresis is an uncommon disease."

Dr. Graves: "One author speaks of paresis as a certificate of intelligence. It certainly is true that the disease occurs with greatest frequency among brain workers."

Paper: "The Treatment of Typhoid Fever," by Dr. F. Paschal. The writer warned against the indiscriminate use of stimulants and also against laxatives. He objected strongly to the use of the Brand bath, and suggested that high temperature in many cases may do good by inhibiting the growth of the specific organism.

This paper called forth an unusual amount of discussion.

Dr. Russ: "I take issue with Dr. Paschal with reference to the use of the cold bath. Like any other therapeutic measure it must, of course, be used with discretion. In cases with persistently high temperature in which the nervous symptoms are prominent, it is certainly indicated. If the duration of the bath and the temperature of the water be properly regulated, and if vigorous massage be practiced during the immersion, reaction will be prompt."

Dr. Kingsley: "As routine treatment I use quinine and strychnine in tonic doses."

Dr. Lankford: "With reference to bowel antisepsis in typhoid fever, I think bile, nature's antiseptic, heads the list in importance, and measures to secure its proper secretion should not be neglected."

Dr. Smith: "I think sufficient prominence has not been given to the use of an abundance of water internally. In many cases much good will result from the use of normal salt solution either by enema or hypodermoclysis."

Dr. Campbell: "A proper disposal of the urine and bowel discharges is of great importance. Too little attention is, as a rule, paid to the preventive treatment of the disease."

Dr. Paschal: "I never use quinine in typhoid fever except in the last stage, when there is great variation in the temperature. Strychnia is of course indicated whenever the heart weakens. I have never observed that the results following the use of the Brand bath are better than are the results following the use of cold in a less heroic way."

The afternoon session was taken up almost entirely by the clinic, a new feature introduced for this meeting at the suggestion of Dr. John S. Lankford, chairman of the program committee. Many interesting cases were presented for study, by far the greater number being furnished by Drs. Burg and Paschal. This clinic was such a decided success that it will probably be repeated on a smaller scale at each of the monthly meetings in future.

The session closed with a paper by Dr. J. S. Lankford on "The Ear and Kidney in Mild Scarlet Fever." [This paper will appear in the JOURNAL soon.—ED.]

Dr. Paschal: "I do not agree with Dr. Lankford that scarlet fever in this climate is milder than elsewhere. Statistics show the mortality here to be about as it is in northern cities."

Dr. Shropshire: "Statistics, then, are misleading. Every practitioner knows that comparatively few cases of scarlet fever are reported to the health department for the good reason that often no physician is called to treat the cases. The mortality from the reported cases, which are the severe ones, is no doubt high."

The evening session was called to order at 8:30.

The following resolutions were unanimously adopted:

"WHEREAS, In the mysterious wisdom of Divine Providence Dr. McPherson Barnitz has been taken from us. Be it

"*Resolved,* That the association has lost a young member of brilliant promise, and the profession of medicine a valued and congenial companion, who was ever ready to render his best service to the poor. Be it further

"*Resolved,* That the association tender expression of deep sympathy to the father, Dr. H. D. Barnitz, and his family, and to the bereaved young widow.

"JOHN S. LANKFORD.
"WM. E. LUTER,
"F. PASCHAL."

PAPER: "Pemphigus, with Report of a Case," by Dr. Russ. Discussion by Drs. C. E. R. and Willis P. King and Dr. Burg.

Dr. Alonzo Garwood, the retiring president, delivered an address, his subject being "Tuberculosis as Seen in Comal County, Texas."

The election of officers for the ensuing year resulted as follows:

President, Dr. William E. Luter; vice-presidents, Dr. H. G. Heaney and Dr. T. T. Jackson; secretary and treasurer, Dr. W. B. Russ; board of censors, Dr. A. Garwood, Dr. James Bartlett, Dr. F. M. Hicks, Dr. R. E. Moss, Dr. F. Paschal.

The banquet held at the Menger Hotel was in every way a success, probably the best banquet, taken all in all, the society has ever had during its twenty-five years of existence.

W. B. Russ, M. D., Secretary.

